# Evaluation of the Performance of an Artificial Intelligence (AI) Algorithm in Detecting Thoracic Pathologies on Chest Radiographs

**DOI:** 10.3390/diagnostics14111183

**Published:** 2024-06-04

**Authors:** Hubert Bettinger, Gregory Lenczner, Jean Guigui, Luc Rotenberg, Elie Zerbib, Alexandre Attia, Julien Vidal, Pauline Beaumel

**Affiliations:** 1 H-CAB, 47 Rue du Rocher, 75008 Paris, France; 2 Radiologie Paris Ouest, 47 Rue du Rocher, 75008 Paris, France; 3 AZmed, 10 Rue d’Uzès, 75002 Paris, France

**Keywords:** artificial intelligence, chest, deep learning, nodules, rayvolve, thorax

## Abstract

The purpose of the study was to assess the performance of readers in diagnosing thoracic anomalies on standard chest radiographs (CXRs) with and without a deep-learning-based AI tool (Rayvolve) and to evaluate the standalone performance of Rayvolve in detecting thoracic pathologies on CXRs. This retrospective multicentric study was conducted in two phases. In phase 1, nine readers independently reviewed 900 CXRs from imaging group A and identified thoracic abnormalities with and without AI assistance. A consensus from three radiologists served as the ground truth. In phase 2, the standalone performance of Rayvolve was evaluated on 1500 CXRs from imaging group B. The average values of AUC across the readers significantly increased by 15.94%, with AI-assisted reading compared to unaided reading (0.88 ± 0.01 vs. 0.759 ± 0.07, *p* < 0.001). The time taken to read the CXRs decreased significantly, by 35.81% with AI assistance. The average values of sensitivity and specificity across the readers increased significantly by 11.44% and 2.95% with AI-assisted reading compared to unaided reading (0.857 ± 0.02 vs. 0.769 ± 0.02 and 0.974 ± 0.01 vs. 0.946 ± 0.01, *p* < 0.001). From the standalone perspective, the AI model achieved an average sensitivity, specificity, PPV, and NPV of 0.964, 0.844, 0.757, and 0.9798. The speed and performance of the readers improved significantly with AI assistance.

## 1. Introduction

Chest radiography is relatively inexpensive and the most frequently performed radiological investigation globally. Chest radiographs play a significant role in screening and diagnosing diseases of cardiothoracic and pulmonary abnormalities [1]. Accurately recognizing abnormalities in chest X-rays (CXR) poses a notable challenge, which is primarily attributed to the scarcity of proficiently trained radiologists and the substantial workload in large healthcare facilities [2]. In addition, the increasing demand for CXRs in the emergency department may directly increase the error rate [3]. Thus, analyzing and reporting chest X-rays is still a challenging and subjective process.

First-generation knowledge-based AI systems depend on the expertise of medical professionals and predefined rules, making them very complicated to develop. In contrast, modern AI uses machine-learning (ML) algorithms to uncover patterns and associations in data [4]. Machine learning (ML) is a branch of AI focused on developing algorithms that can identify patterns, make predictions, and automate decisions. As ML models are exposed to data that are more heterogeneous over time, their performance continues to improve. A class of ML models called artificial neural networks (ANNs), which mimic the brain’s neural networks, consist of layers of interconnected nodes (that resemble the neurons in the brain), that learn to recognize patterns. The connections between these nodes have weights that adjust as the network learns from data, improving its performance over time. ANNs are particularly good at identifying patterns and making complex decisions based on input data. Deep learning (DL), a subset of ML, uses multi-layered ANNs known as deep neural networks to automatically learn and extract complex features from large datasets. This allows DL to excel at tasks like image and speech recognition [5].

The use of AI in radiology has revolutionized the way healthcare professionals diagnose and treat patients. Since 2020 and the start of the SARS-CoV-2 pandemic, the use of artificial intelligence (AI) in radiology has increasingly captured attention in the clinical, research, and commercial domains within the field of medical imaging [6,7,8]. As the pandemic overwhelmed healthcare systems worldwide, the urgent need for rapid and accurate diagnosis pushed the adoption of AI technologies to new heights that will continue to benefit the field of radiology long after the pandemic subsides. In 2022, Kufel et al. concluded in their systematic review that, with appropriate modifications, the developed AI tools could be utilized to diagnose other diseases related to chest organs in the future [9]. This hypothesis has now been confirmed, as multiple deep-learning (DL) technologies are currently being used to assist in triaging and detecting different chest anomalies, including pneumonia, pleural effusion, pulmonary nodules, tuberculosis, and pneumothorax [10,11,12,13,14,15,16,17,18]. Rayvolve, developed by AZmed (Paris, France) is a DL-based AI tool developed in a multi-centric environment. Rayvolve has been a CE-marked class IIa under Regulation (EU) 2017/745 (MDR). The Rayvolve algorithm is an ensembling algorithm composed of five object-detection models based on the RetinaNet [19] architecture with the same VGG-16 backbone [20] for detecting multiple pathologies, namely consolidation, pleural effusion, pneumothorax, acute pulmonary edema, cardiomegaly, and pulmonary nodules on chest radiographs.

AI models are significantly enhancing the capabilities of radiologists by improving diagnostic accuracy, streamlining workflow, prioritizing critical cases, and, ultimately, enhancing patient care. It is expected that an AI-based computer-aided diagnosis (CAD) system could effectively support radiologists and reduce the misdiagnosis rate in interpreting CXR [21]. In this context, exploring the ways in which AI is augmenting chest radiography offers profound insights into the transformative potential of technology in modern healthcare practices. However, it is essential to rigorously assess its effectiveness to ensure safe and efficient use in clinical practice.

Since limited evidence exists regarding the influence of AI on reader efficiency, particularly concerning the time it takes for readers to finalize their reports [10], we hypothesized that Rayvolve, when used as a concomitant read, will improve the reader’s performance in diagnosing anomalies on standard chest radiographs and will reduce the time taken to read the X-rays. Furthermore, we wanted to evaluate the standalone performance of Rayvolve in detecting thoracic pathologies on chest radiographs of patients to validate its performance in the real-world clinical setting.

## 2. Materials and Methods

This study was conducted and reported according to the transparent reporting of a multivariable prediction model for individual prognosis or diagnosis (TRIPOD) guideline [22]. The TRIPOD checklist for prediction-model development and validation is reported in Appendix A Table A1.

### 2.1. Study Design

**Figure 1:**
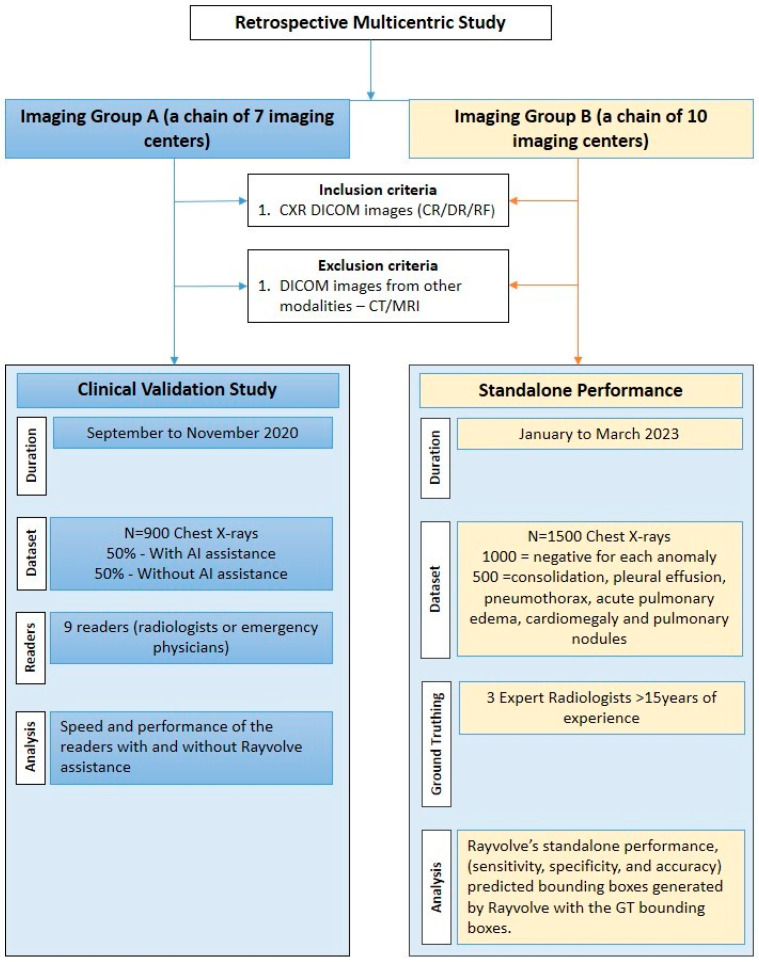
Flowchart of the study procedure for clinical validation and standalone performance evaluation of Rayvolve.

A retrospective multi-reader, multi-centric (MRMC) study was conducted in 2 phases. In the first phase, a clinical validation study was performed where nine readers, either radiologists or emergency physicians, independently reviewed nine hundred chest X-rays. In phase 2, the standalone performance of the AI model was evaluated. The study was registered with the French National Security Agency of Medicines and Health Products (ANSM) under the number 2020-A02639-30. Informed consent was waived because of the retrospective nature of the study. A flowchart of the study procedure is shown in Figure 1.

### 2.2. Algorithm Development

#### 2.2.1. Algorithm Design and Function

**Figure 2:**
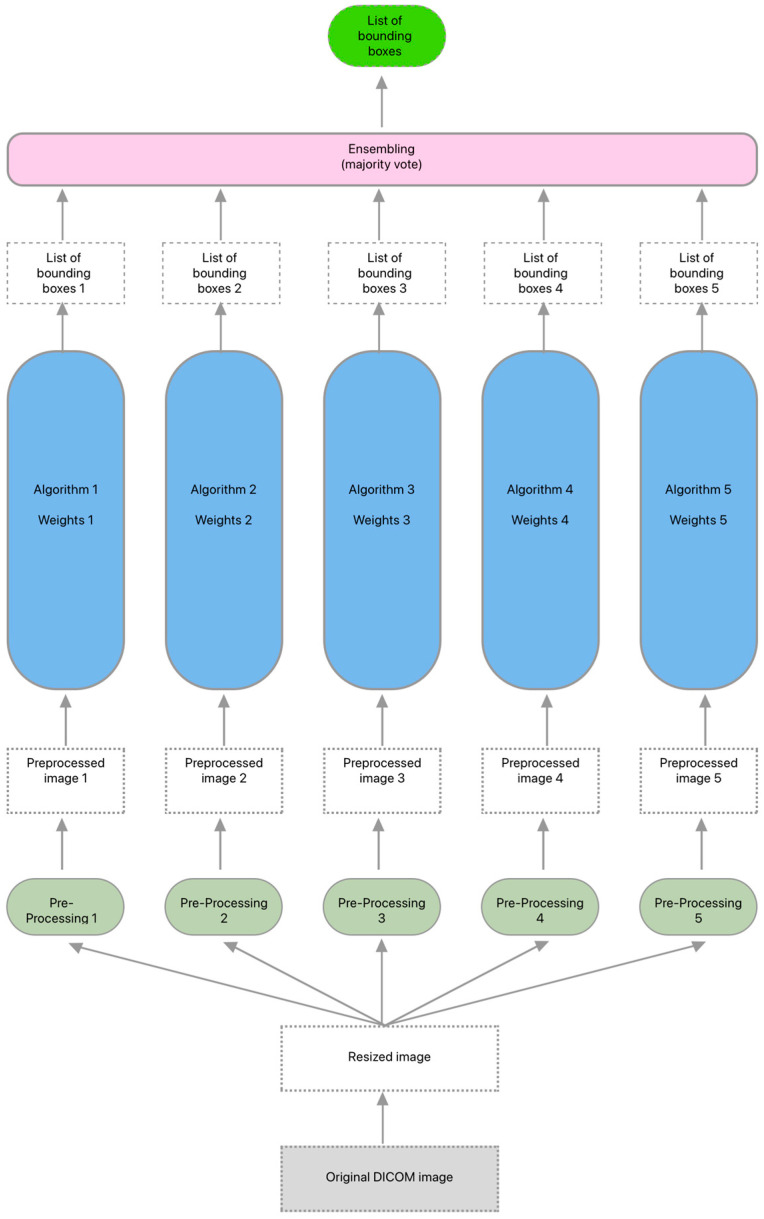
Dataset used to train Rayvolve algorithm.

The Rayvolve algorithm was designed to detect radiological signs of multiple pathologies, namely consolidation, pleural effusion, pneumothorax, acute pulmonary edema (APE), cardiomegaly, and pulmonary nodules on chest X-rays. The Rayvolve algorithm is an ensembling algorithm composed of 5 object-detection models based on RetinaNet [19] architecture with the same VGG-16 backbone [20]. The object-detection models were composed of trainable convolutional layers, non-trainable max-pooling layers, and trainable batch-normalization layers. Each object-detection model outputs a list of bounding boxes (this list could be empty if no abnormalities were detected by the object-detection model) with their respective confidence scores and their respective pathology classifications. To choose the final list of bounding boxes, AZmed used a majority vote between the predicted bounding boxes (the vote was done only on bounding boxes of the same pathology) for each of the five models. To retain, a bounding box should have a non-null intersection, with at least two other bounding boxes from two other different object-detection models. The list of bounding boxes outputted by the ensembling algorithm was the bounding boxes that have been outputted by at least three object-detection models, and thus, an overlapping between three bounding boxes was necessary to keep one final bounding box (dependent on the pathology). Between the overlapping bounding boxes, AZmed kept the one with the highest score. Each object-detection model (called Model X, with X as the index on Figure 2) has the same right to vote. During the ensembling, a last step was done to filter the bounding boxes with a score lower than a determined threshold for each pathology. This threshold was determined using a validation dataset (without any intersection with the training dataset).

#### 2.2.2. Transfer Learning and Databases

The radiography databases used for transfer learning were:

- Model one has been pre-trained on COCO [23];
- Model two has been pre-trained on CheXpert [24];
- Model three has been pre-trained on COCO and CheXpert;
- Model four has been pre-trained on MIMIC [25];
- Model five has been pre-trained on COCO and MIMIC.

As the initialized weights were different from each other between the models, the final weights were different. Thus, the predictions made by each model could differ.

#### 2.2.3. Dataset

The dataset used to train the Rayvolve algorithm is composed of labeled chest radiographs (see Figure 2). AZmed used the exact same dataset for every object-detection model. The chest radiographs were collected from multiple centers (the different medical-imaging centers included public hospitals, private clinics, generalist medical-imaging centers, and musculoskeletal medical-imaging centers) from different countries (France, Israel, Germany, Switzerland, Belgium, United Kingdom, Brazil, and Nigeria) in order to have the largest diversity and variety to allow the Rayvolve algorithm to have a high generalization ability.

This dataset was divided into three distinct sub-datasets: (a) a training set composed of 50,000 chest radiographs, with at least one abnormality from the list of pathologies below; (b) a validation set composed of 10,000 chest radiographs (4000 radiographs with at least one abnormality from the list of pathologies below and 6000 radiographs without any abnormality); and (c) a test set composed of 2000 chest radiographs (1000 chest radiographs with at least one abnormality from the list of pathologies below and 1000 radiographs without any abnormality).

#### 2.2.4. Algorithm Training

The training of each object-detection algorithm was done separately. During the training, the goal was to minimize the cost, i.e., the sum of one classification focal loss and one regression smooth-L1 loss. During the training, a batch of eight pre-processed images were passed to the algorithm at each iteration. The training was composed of 50,000 iterations.

#### 2.2.5. Threshold Definition and Utility of the Validation Set

During the training of each model, the aim was to adjust the learning rate only when the optimizer could not improve the results. After each epoch, the performance on the validation set was computed. The learning rate was divided by two if the results were not improved. At the end of training each model, AZmed chose the threshold for each pathology regarding the confidence scores of the bounding boxes predicted by the model. AZmed chose the optimal threshold to have the higher performance (the metrics are the sensitivity, specificity, and mAP) on the validation dataset.

### 2.3. Ground Truth Labelling for Thoracic Abnormalities

The CXRs were independently interpreted and annotated by three radiologists with over 15 years of experience to establish the reference standard.

Each radiologist performed the readings to determine the absence or presence of a radiological sign of abnormality, namely consolidation, pleural effusion, pneumothorax, APE, cardiomegaly, and pulmonary nodules. The radiologists annotated the abnormality by drawing a bounding box of minimal size to enclose the entire radiological sign. The pathological localization of ground truth (GT) was then defined as the average between the radiologists’ bounding boxes.

Ground truth for the presence/absence of a radiological sign was defined as the majority opinion of at least two of the three clinicians participating in the ground truthing process. An agreement between the bounding boxes drawn by two (or three) radiologists was considered when there was an overlap between the bounding boxes for the same radiological sign (i.e., the Jaccard distance between the bounding boxes of each radiologist is non-null; thus, the overlapping is defined by at least one pixel of overlap).

Rayvolve’s standalone performance was evaluated by its sensitivity, specificity, and accuracy. These metrics were calculated by comparing the predicted bounding boxes generated by Rayvolve with the GT bounding boxes. A predicted bounding box overlapping with a GT bounding box was classified as a true positive (TP); otherwise, it was classified as a false positive (FP). Conversely, if no predicted bounding box was present, it was counted as a true negative (TN). Instances where the ground truth did not overlap with a predicted bounding box were counted as false negatives (FN).

### 2.4. Sample-Size Calculation

To compute the required sample size for the comparison of the area under an ROC curve with a null hypothesis value, AZmed took into account the required significance level and power of the study. For this, AZmed used the following inputs: Type I error—Alpha = 0.05, Type II error—Beta = 0.20, hypothesized AUC = 0.80. There is an estimation of a minimum of 26 radiographs per anomaly (with at least 13 positive radiographs and 13 negative radiographs). Thus, AZmed estimated a minimum of 26 radiographs per anomaly (with at least 13 positive radiographs and 13 negative radiographs).

### 2.5. Clinical Validation Study

In this MRMC study, nine hundred chest X-rays from imaging group A (a chain of seven imaging centers) located in Marne-la-Vallée, France, were investigated from September 2020 to November 2020. Only chest X-ray images (CR/DR/RF) in DICOM format with posterior–anterior (PA) or anterior–posterior (AP) views or lateral views were included. The exclusion criteria included DICOM images coming from other modalities, like MRI, CT, mammography, or ultrasound. CXRs with any missing data were also excluded.

The chest X-rays were randomly selected to evaluate the impact of Rayvolve when used as a concurrent read on readers’ performance in diagnosing anomalies on standard radiographs with and without AI aid. Nine radiologists or emergency physicians independently read the X-rays. Two reading sessions were planned. In the first reading session, the readers read 450 CXR images and made their diagnosis not aided by Rayvolve. In the second reading session, the readers read the other set of 450 CXR images and made their diagnosis aided by Rayvolve.

The speed and performance of the readers while conducting CXR analysis with and without AI aid were evaluated in terms of AUC, sensitivity, specificity, and mean time spent in the analysis of an X-ray. The specificity per anomaly was defined as the proportion of CXR with no anomaly indicated by the readers using a bounding box among all the normal CXRs. The sensitivity per anomaly was defined as the proportion of anomalies correctly identified by the readers among all the anomalies. The average sensitivity and specificity were then calculated.

### 2.6. Evaluation of AI Standalone Performance

The standalone performance of Rayvolve was assessed on 1500 CXRs acquired from imaging group B (a chain of 10 imaging centers), located in the west of Paris from January 2023 to March 2023. Five hundred CXRs with the presence of a radiological sign of abnormality for each thoracic pathology, including consolidation, pleural effusion, pneumothorax, APE, cardiomegaly, and pulmonary nodules, were included. In addition, one thousand CXRs with no sign of abnormality for each thoracic pathology mentioned above were also included.

The radiographs were assessed by Rayvolve and then by expert radiologists to detect the presence of thoracic pathologies. The results of Rayvolve were compared to those of a consensus of three expert radiologists constituting a GT to evaluate the relative effectiveness of AI in detecting thoracic pathologies. Rayvolve categorized each analyzed image into one of four groups: FP, FN, TP, and TN.

Evaluation of the AI algorithm’s performance was conducted using well-established performance metrics, such as sensitivity, specificity, positive predictive value (PPV), negative predictive value (NPV), and the receiver operating characteristic (ROC) curve.

The differences in average time spent on analyzing a CXR, values of AUC, specificity, and sensitivity to evaluate the performance results of the readers with and without Rayvolve (with AI vs. without AI) were analyzed using a *t*-test with 95% CI. The significance level was set to *p* < 0.05. The analysis was done using Python, version 3.10, programming software. Bootstrap resampling was employed to estimate 95% CIs for the AUC (2000 bootstrap samples). For sensitivity and specificity, 95% CIs were calculated using Wilson’s method.

## 3. Results

### 3.1. Clinical Validation Study

The clinical validation study was performed on 900 CXR images. There was a total of 640 CXRs with no anomaly and 260 CXRs with an anomaly (consolidation = 77, pleural effusion = 60, pneumothorax = 32, cardiomegaly = 16, OAP = 37, and pulmonary nodule = 43).

**Figure 3:**
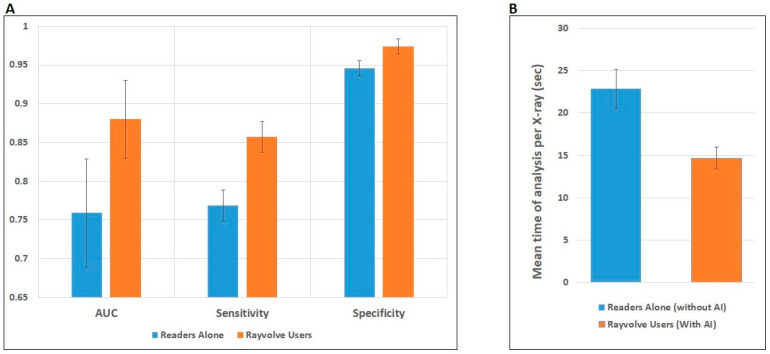
Detection performance of the readers with and without Rayvolve (**A**) AUC, sensitivity, and specificity, (**B**) represent the mean time of analysis per X-ray.

The performance results of the readers with and without Rayvolve aid are reported in Table 1. The average values of AUC across the readers were significantly increased by 15.94%, with Rayvolve-assisted reading compared to unaided reading (0.88 ± 0.01 vs. 0.759 ± 0.07, *p* < 0.001). Time taken to read the chest X-rays decreased significantly, by 35.81%, with the use of Rayvolve (14.7 ± 1.3 vs. 22.9 ± 2.3, *p* < 0.001). Also, the average values of sensitivity and specificity across the readers increased significantly, by 11.44% and 2.95%, with Rayvolve-assisted reading compared to unaided reading (0.857 ± 0.02 vs. 0.769 ± 0.02, *p* < 0.001 and 0.974 ± 0.01 vs. 0.946 ± 0.01, *p* < 0.001, respectively) (see Figure 3).

### 3.2. AI Standalone Performance Evaluation

A total of 1500 chest radiographs were used in assessing the standalone performance of Rayvolve (mean age, 49.9 years ± 23 [standard deviation]; female = 818 (54.5%) and male = 682 (45.5%)). The multicentric dataset was obtained from GMM/Primax (17%) Fujifilm (11%), Shimadzu (38%), Canon (28%), and Medecom (6%).

**Figure 4:**
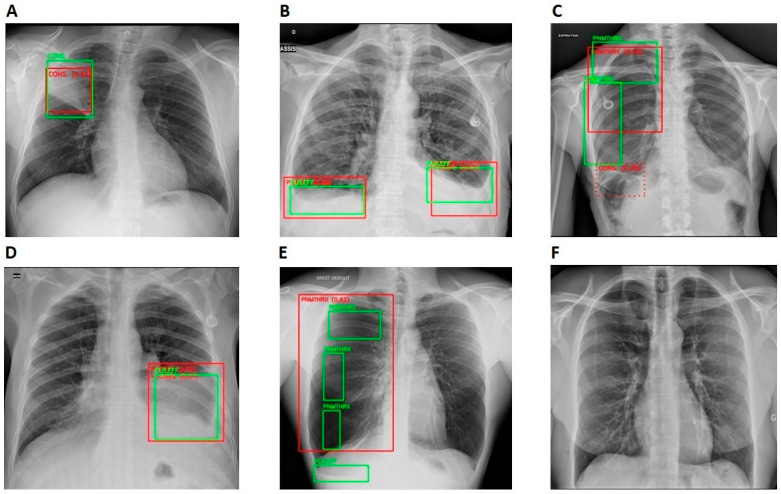
Standalone performance of Rayvolve examples. Green and red boxes represent ground truths and the AI model predictions. (**A**) Rayvolve correctly identified consolidation (TP). (**B**) Rayvolve correctly identified pleural effusion (TP). (**C**) The AI model correctly identified pneumothorax on the right lung. However, it incorrectly detected consolidation on the right lung (TP for pneumothorax and FP for consolidation). (**D**) The AI model correctly identified pleural effusion on the left lung. However, it incorrectly detected consolidation on the left lung (TP for pleural effusion and FP for consolidation). (**E**) Rayvolve correctly identified pneumothorax. However, it missed pleural effusion on the right lung (TP for pneumothorax and FN for pleural effusion). (**F**) The AI model correctly identified a normal scan (TN).

The AI model achieved an average sensitivity, specificity, and PPV and NPV of 0.964, 0.844, 0.757, and 0.9798, across all the centers, respectively. The highest false-positive rate (FPR) was observed in the nodules (20.1%), followed by the pneumothorax (18.8%) and the cardiomegaly (16.1%). Consolidation had the lowest FPR of 11.4% but the highest false-negative rate (FNR) of 7%, compared to other anomalies. Nodules had the lowest FNR of 1.2% followed by cardiomegaly (2.2%). The distribution of overlap of TP, TN, FP, and FN cases between Rayvolve and the reference standard is shown in Table 2. The performance metrics per anomaly are shown in Table 3. Examples of TP, TN, FP, and FN CXR studies are shown in Figure 4.

**Figure 5:**
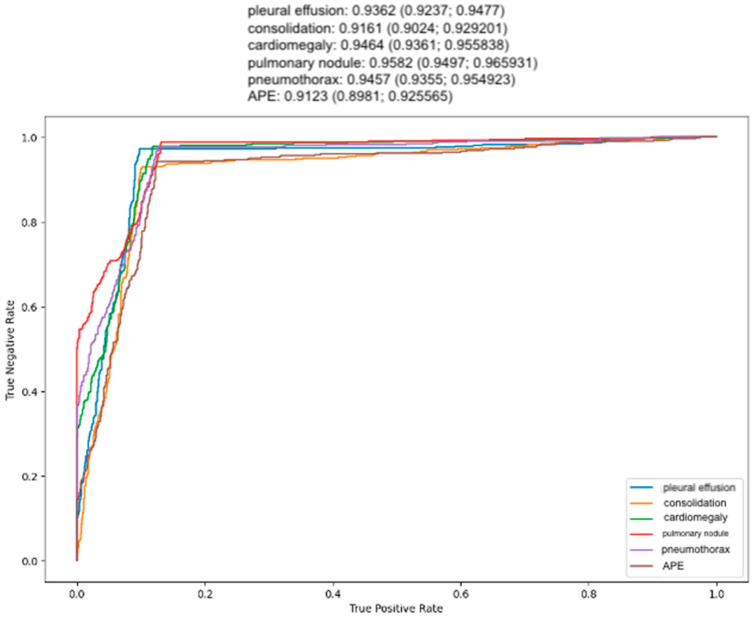
Receiver operating characteristic (ROC) curves of Rayvolve standalone performance in detecting the thoracic anomalies.

From the standalone performance perspective, Rayvolve identified the target thoracic anomalies with sensitivities of 0.93 to 0.988, specificities of 0.795 to 0.886, NPVs of 0.962 to 0.9925, and PPVs of 0.707 to 0.803. Rayvolve achieved an overall AUC of 0.9358 [95% CI, 0.921–0.950], sensitivity of 0.964 [95% CI, 0.946–0.9826] and specificity of 0.8435 [95% CI, 0.8155–0.87147]. To add on, the DL algorithm showed high diagnostic accuracy for detecting lung nodules (sensitivity = 0.988, specificity = 0.795, AUROC = 0.958 [95% CI, 0.9497–0.9659]) compared to the other anomalies. In addition, Rayvolve showed a low diagnostic accuracy for detecting APE (sensitivity = 0.942, specificity = 0.859, AUROC = 0.9123 [95% CI, 0.8981–0.9255]). The standalone performance of Rayvolve in detecting each target anomaly against the radiologist’s performance is illustrated in Figure 5. Compared with the reference standard set up by three expert radiologists, the standalone performance of Rayvolve was 97.2% (486/500) for detecting pleural effusion, 93% for consolidation (465/500), 97.8% for cardiomegaly (489/500), 98.8% for nodules (494/500), 97.6% for pneumothorax (488/500), and 94.2% for APE (471/500).

## 4. Discussion

In this retrospective study, the diagnostic performance of the readers in detecting the thoracic anomalies on standard chest radiographs using a commercially available algorithm, Rayvolve, was evaluated.

A clinical validation study showed that chest X-ray reporting using the aid of Rayvolve significantly improved the speed and performance of the readers compared to CXR reporting without AI assistance. Interestingly, the study reported that the time taken to report the CXR reduced significantly by 35.8% with AI aid, which is an 8.2 s saving per exam. The significant reduction in the average reporting time of a CXR may be attributed to several reasons. Since the AI tool can automatically detect and highlight abnormalities using a bounding box on the CXRs, this might have saved time for the readers by drawing their attention to areas that may need further examination. Having AI aid can increase a reader’s confidence in their interpretations, leading to faster decision-making and reducing the time spent deliberating over findings.

Moreover, improvement in accuracy, as shown by a 15.94% increase in the AUC for readers using AI assistance compared to those without it (0.88 vs. 0.759), was noted. Additionally, their sensitivity improved by 11.44% and their specificity by 2.95%. The results are comparable to the findings from another multi-center multi-reader study that reported significantly higher AUCs with AI aid than without AI assistance (0.886 vs. 0.861, *p* = 0.003) for detecting thoracic pathologies on CXRs [17]. Similarly, the study by Ej et al. [26] has also shown an improvement in the performance of physicians with AI aid. Therefore, AI can be used in concurrent reading to improve the speed and performance of radiologists or emergency practitioners.

Chest X-rays often serve as a frontline screening tool for detecting a variety of thoracic and pulmonary abnormalities. The DL algorithm’s standalone performance demonstrated that the tool could perform with high accuracy and sensitivity, with an overall AUC of 0.9358 [95% CI, 0.921–0.950], sensitivity of 0.964 [95% CI, 0.946–0.9826], and specificity of 0.8435 [95% CI, 0.8155–0.87147], confirming its robustness in a clinical setting. Although Rayvolve’s standalone performance showed similar sensitivity to that of radiologists, its specificity was notably lower. The threshold used to classify cases as positive or negative by the AI tool could have influenced its sensitivity and specificity. A threshold set too low will increase sensitivity but decrease specificity, leading to more FPs. Adapting the threshold for particular locations can be done to overcome domain generalization issues. In the real world, such as a clinical setting with increased patient load and radiologist staffing shortages, deploying an AI tool in the clinical workflow with maximizing sensitivity is crucial to ensure that abnormal findings are not missed during initial screening.

The mean NPV of 0.9798 and mean PPV of 0.757 proves the efficacy of Rayvolve in ruling out the true-negative cases, thus allowing prioritization of the abnormal CXRs with anomalies. However, the reduced PPV can be ascribed to both a higher FP and the low prevalence of the thoracic anomalies within the dataset. Additionally, detecting anomalies on CXRs could be challenging due to the presence of overlapping structures and subtle abnormalities in the thoracic region, resulting in higher FP. This could lead to potential false alarms, unnecessary interventions, or follow-ups. Minimizing FN and FP is crucial for improving the diagnostic accuracy of the AI model. This could be achieved by continuous learning of the AI model and by validating the model on external datasets from different populations and geographical areas to ensure robustness and generalizability. The more diverse the data, the better would be the ability of the model to identify pathologies, reducing the likelihood of missing rare pathologies or misclassifying normal cases. The authors would rework the images detected as FP/FN and would place particular emphasis on the FN used in the continuous learning work.

A high AUC score of 0.9358 suggests that the AI model has a strong ability to differentiate between distinct types of abnormalities. Therefore, a high AUC score for Rayvolve indicates its capability to accurately find and categorize different abnormalities in chest X-rays. However, among all the abnormalities, APE and consolidation achieved the lowest AUC scores of 0.9123 and 0.9161, respectively. This may be attributed to the high number of FN cases, i.e., twenty-nine for APE and thirty-five for consolidation.

The current study had limitations. First, the results of the study are based on the data collected from multiple centers within Paris, France, so the generalizability across other geographical settings is uncertain. Secondly, the authors did not perform any subgroup analysis to evaluate how the AI model performed across different confounding factors (age, sex, and manufacturer). Third, this is a non-exhaustive AI algorithm that was trained to detect only six pathologies.

To our knowledge, this is the first study to evaluate the performance of a commercial AI tool, which in the future can aid in triaging X-rays, prioritizing those that require immediate attention or further review, and allowing radiologists to focus their time and expertise where it is most needed. The authors recommend performing a randomized clinical trial using a prospective design to evaluate the AI model’s performance in a more rigorous setting. This will help establish the AI model’s credibility and evaluate its stability, reliability, and impact on clinical outcomes in real-world scenarios. Further, future studies should focus on retraining the model using diverse data to identify other thoracic anomalies and to evaluate and compare its performance with that of radiologists.

## 5. Conclusions

The findings of this study can lead to the adoption and integration of the AI tool in a real-world clinical setting. This will help automate and streamline the workflow in the radiology department, thus reducing the time and workload for radiologists. In conclusion, an AI tool, when used as a concomitant read, can significantly improve the performances of radiologists or emergency practitioners in detecting consolidation, pleural effusion, pneumothorax, APE, cardiomegaly, and pulmonary nodules and can save the time taken to analyze chest radiographs.

## Figures and Tables

**Table 1:** Descriptive statistics of the main outcomes with and without AI assistance.

	Readers Alone (without AI Aid)				Rayvolve Users (with AI Aid)				*t*-Test
	Mean	SD	Var	CI (95%)	Mean	SD	Var	CI (95%)	*p*-Value
**Time (s)**	22.9	2.3	5.4	1.503	14.7	1.3	1.7	0.849	<0.001 *
**AUC**	0.759	0.07	0.01	0.046	0.88	0.05	0.01	0.033	<0.001 *
**Sensitivity**	0.769	0.02	0	0.013	0.857	0.02	0	0.013	<0.001 *
**Specificity**	0.946	0.01	0	0.007	0.974	0.01	0	0.007	<0.001 *

* Significant at *p* < 0.05; SD: standard deviation; Var: variance; CI: confidence interval.

**Table 2:** Distribution of overlap of true-positive, true-negative, false-positive, and false-negative cases between Rayvolve and the reference standard in thoracic radiographs.

	TP	FN	FP	TN
Pleural effusion	486	14	130	870
Consolidation	465	35	114	886
Cardiomegaly	489	11	161	839
Nodules	494	6	205	795
Pneumothorax	488	12	188	812
APE	471	29	141	859

**Table 3:** Rayvolve performance metrics per anomaly.

Anomaly	Sensitivity	Specificity	NPV	PPV	AUC [95% CI]
Pleural effusion	0.972	0.87	0.984	0.789	0.9362 (0.9237–0.9477)
Consolidation	0.93	0.886	0.962	0.803	0.9161 (0.9024–0.9292)
Cardiomegaly	0.978	0.839	0.987	0.752	0.9464 (0.9361–0.9558)
Nodules	0.988	0.795	0.9925	0.707	0.9582 (0.9497–0.9659)
Pneumothorax	0.976	0.812	0.9854	0.7219	0.9457 (0.9355–0.9549)
APE	0.942	0.859	0.9673	0.7696	0.9123 (0.8981–0.92556)

## Data Availability

Data will be available upon request from the corresponding author.

## Appendix A

**Table A1:** TRIPOD Checklist.

Section/Topic	Item		Checklist Item	Page
**Title and abstract**				
Title	1	D; V	Identify the study as developing and/or validating a multivariable prediction model, the target population, and the outcome to be predicted.	1
Abstract	1	V	Provide a summary of objectives, study design, setting, participants, sample size, predictors, outcome, statistical analysis, results, and conclusions.	1
**Introduction**				
Background and objectives	3a	V	Explain the medical context (including whether diagnostic or prognostic) and rationale for developing or validating the multivariable prediction model, including references to existing models.	1, 2
	3b	V	Specify the objectives, including whether the study describes the development or validation of the model or both.	2
**Methods**				
Source of data	4a	V	Describe the study design or source of data (e.g., randomized trial, cohort, or registry data), separately for the development and validation of data sets, if applicable.	2
	4b	V	Specify the key study dates, including the start of accrual; end of accrual; and, if applicable, end of follow-up.	6, 7
Participants	5a	V	Specify key elements of the study setting (e.g., primary care, secondary care, and general population) including the number and location of centers.	6, 7
	5b	V	Describe eligibility criteria for participants.	n/a
	5c	V	Give details of treatments received, if relevant.	n/a
Outcome	6a	V	Clearly define the outcome that is predicted by the prediction model, including how and when assessed.	6,7
	6b	V	Report any actions to blind assessment of the outcome to be predicted.	n/a
Predictors	7a	V	Clearly define all predictors used in developing or validating the multivariable prediction model, including how and when they were measured.	6, 7
	7b	V	Report any actions to blind assessment of predictors for the outcome and other predictors.	n/a
Sample size	8	V	Explain how the study size was arrived at.	6
Missing data	9	V	Describe how missing data were handled (e.g., complete-case analysis, single imputation, and multiple imputation) with details of any imputation method.	6
Statistical analysis methods	10a	V	Describe how predictors were handled in the analyses.	7
	10b	V	Specify the type of model, all model-building procedures (including any predictor selection), and methods for internal validation.	3, 4, 5, 6, 7
	10c	V	For validation, describe how the predictions were calculated.	7
	10d	V	Specify all measures used to assess model performance and, if relevant, to compare multiple models.	7
	10e	V	Describe any model updating (e.g., recalibration) arising from the validation, if conducted.	7
Risk groups	11	V	Provide details on how risk groups were created, if conducted.	n/a
Development vs. validation	12	V	For validation, identify any differences from the development data in setting, eligibility criteria, outcome, and predictors.	n/a
**Results**				
Participants	13a	V	Describe the flow of participants through the study, including the number of participants with and without the outcome and, if applicable, a summary of the follow-up time. A diagram may be helpful.	3
	13b	V	Describe the characteristics of the participants (basic demographics, clinical features, and available predictors), including the number of participants with missing data for predictors and outcome.	7, 8
	13c	V	For validation, show a comparison with the development data of the distribution of important variables (demographics, predictors, and outcome).	n/a
Model development	14a	V	Specify the number of participants and outcome events in each analysis.	7, 8
	14b	V	If done, report the unadjusted association between each candidate predictor and outcome.	n/a
Model specification	15a	V	Present the full prediction model to allow predictions for individuals (i.e., all regression coefficients and model intercept or baseline survival at a given time point).	n/a
	15b	V	Explain how to use the prediction model.	
Model performance	16	V	Report performance measures (with CIs) for the prediction model.	8, 9
Model-updating	17	V	If done, report the results from any model updating (i.e., model specification and model performance).	n/a
**Discussion**				
Limitations	18	V	Discuss any limitations of the study (such as a nonrepresentative sample, few events per predictor, and missing data).	12
Interpretation	19a	V	For validation, discuss the results with reference to performance in the development data and any other validation data.	n/a
	19b	V	Give an overall interpretation of the results, considering objectives, limitations, results from similar studies, and other relevant evidence.	11, 12
Implications	20	V	Discuss the potential clinical use of the model and its implications for future research.	12
**Other information**				
Supplementary information	21	V	Provide information about the availability of supplementary resources, such as the study protocol, Web calculator, and data sets.	12, 13
Funding	22	V	Give the source of funding and the role of the funders for the present study.	12
